# Rapid Analysis of Glibenclamide Using an Environmentally Benign Stability-Indicating RP-HPLC Method 

**Published:** 2014

**Authors:** Nazrul Haq, Fars Kaed Alanazi, Ibrahim Abdullah Alsarra, Faiyaz Shakeel

**Affiliations:** a *Center of Excellence in Biotechnology Research (CEBR), King Saud University, Riyadh-11451, Saudi Arabia. *; b *Department of Pharmaceutics, College of Pharmacy, King Saud University, P.O. Box 2457, Riyadh-11451, Saudi Arabia. *; c *Kayyali Chair for Pharmaceutical Industry, Department of Pharmaceutics, College of Pharmacy, King Saud University, P.O. Box 2457, Riyadh-11451, Saudi Arabia. *

**Keywords:** Glibenclamide, Green RP-HPLC, Nanoemulsion, UV detection, Validation

## Abstract

An environmentally benign RP-HPLC approach for rapid analysis of glibenclamide in pure form, developed nanoemulsion and commercial tablets was developed and validated in present investigation. The green chromatographic identification was performed on Lichrosphere 250 X 4.0 mm RP C_8 _column having a 5 μm packing as a stationary phase using a combination of ethanol: methanol (50:50 % v/v) as a mobile phase, at a flow rate of 1.0 mL/min with UV detection at 245 nm. The proposed method was validated for linearity, selectivity, accuracy, precision, robustness, sensitivity and specificity as per international conference on harmonization (ICH) guidelines. The utility of proposed method was verified by assay of glibenclamide in developed nanoemulsion and commercial tablets. The proposed method was found to be satisfactory in terms of selectivity, precision, accuracy, robustness, sensitivity and specificity. The content of glibenclamide in developed nanoemulsion and commercial tablets was found to be 100.50 % and 99.15 % respectively. The proposed method successfully resoled glibenclamide peak in the presence of its all type of degradation products which indicated stability-indicating property of the proposed method. These results indicated that the green chromatographic method could be successfully employed for routine analysis of glibenclamide in pure drug and various commercial formulations.

## Introduction

The pursuit in the field of green chemistry is growing dramatically and is becoming a grand challenge for chemists (to develop new products, processes and services that achieve the necessary social, economical and environmental objectives) due to an increased cognizance for environmental safety, checking environmental pollution, sustainable industrial ecology and cleaner production technologies worldwide. Many solvents used in the analytical methodologies are volatile organic compounds (VOCs), hazardous air pollutants (HAPs), flammable, toxic and/or carcinogenic [*e.g*., the majority of analytical methods certified by the US Environmental Protection Agency (EPA) and Food and Drug Administration (FDA) use corrosive and toxic chemicals], with no other options currently available (1). They also pose serious environmental, health, and safety (EHS) concerns, including human and eco-toxicity issues, process safety hazards, and waste management issues. 

**Figure 1:**
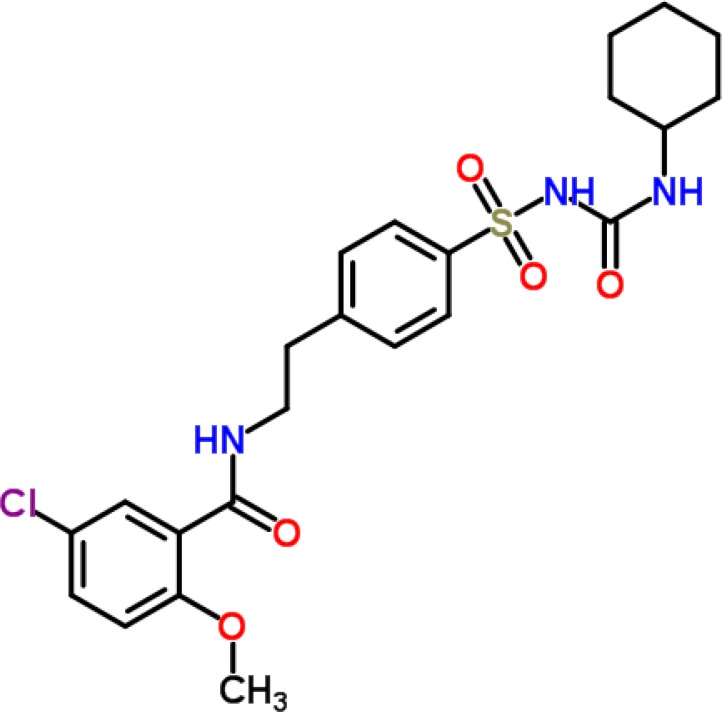
Chemical structure of glibenclamide

Glibenclamide (5-chloro-N-(2-{4- [(cyclohexylcarbamoyl)sulfamoyl]phenyl} ethyl)-2-methoxybenzamide, Figure 1), also known as glyburide, is a second generation sulfonylurea that is extensively used for the treatment of type 2 diabetes mellitus as well as in gestational diabetes (2). An extensive literature survey revealed that many analytical methods have been reported for the analysis of glibenclamide either alone or in combination with other hypoglycemic agents in pure drugs, marketed tablets and biological fluids (like plasma, serum and urine). Several high performance liquid chromatography (HPLC) methods were reported for analysis of glibenclamide alone or in combination with other anti-diabetic agents in pure drugs, tablet dosage forms or biological fluids (3-18). Some spectrophotometric methods have also been developed for the quantitative analysis of glyburide alone or in combination with other sulfonylurea in pure drugs or pharmaceutical formulations (19, 20). Capillary zone electrophoretic (CZE) methods were also used for quantitative analysis of glibenclamide in tablet dosage forms (21, 22). Among all the methods employed for the analysis of glibenclamide, the most commonly employed method for the analysis of glyburide is HPLC either alone or in combination with mass spectrometry because concentrations of glyburide clinically rarely exceed 400 ng/mL, so other methods such as fluorometric and spectrophotometric (19, 20) lack the specificity and sensitivity necessary for the detection of glyburide in plasma whereas gas chromatographic (GC) methods (23) require the preparation of volatile and thermally stable derivatives introducing a time-consuming derivatization step. 

On analyzing the scientific articles of the past several years available for the assay of the glibenclamide by HPLC, it becomes clear that organic and mixed aqueous-organic solvent systems have been found most useful for its analysis. Majority of these solvents are volatile organic compounds and poses a threat to the environment as they form low-level ozone and smog through free radical air-oxidation processes. The main goal of the green analytical chemistry is to take into consideration the amount and the toxicity of reagents consumed, and, consequently, the volume and the toxicity of wastes generated during method development and selection, in this way reducing the environmental impact of the activities of analytical chemistry (24, 25). Particular attention should be paid to solvents as 80% of waste generated during manufacture of a typical active pharmaceutical ingredient (API) is related to solvent use. Green solvents or biosolvents are more environment-friendly alternative to petrochemical solvents, which are derived from processing of agricultural crops, for example, ethyl acetate and ethanol. 

To our surprise, in spite of several favorable features such as non-toxicity, non-inflammability, non-aggressiveness, high biodegradability and cost effectiveness, full potential of environmentally benign solvents (and their combinations) as the eluents for HPLC analysis, in general and for drugs/pharmaceuticals in particular, has not been exploited extensively (26- 28). Nevertheless, some green analytical methods such as reflectometric, spectrophotometric and chromatographic methods have been reported for analysis of some drugs (29-32). 

To our knowledge, stability-indicating green reverse phase HPLC (RP-HPLC) method using environmentally benign eluent ethanol in combination with methanol (50:50 v/v) has not been reported in the literature for analysis of anti-diabetic drug glibenclamide in pharmaceutical formulations and biological fluids. Therefore, the aim of present study was to develop and validate a facile, cost effective, rapid, selective, precise, accurate, robust and stability-indicating green RP-HPLC method coupled with UV detection for rapid analysis of glyburide in developed nanoemulsion and marketed tablets utilizing a combination of methanol and ethanol (50:50 v/v) as mobile phase and isocratic elution, taking into considerations a variety of international conference on harmonization (ICH) recommended test conditions (33). The novelty of this method is that it used an environmentally friendly mobile phase (ethanol) with C_8_ RP-HPLC column for rapid analysis of glibenclamide. The developed method would also be utilized for studying the stability of glibenclamide in various commercial and in-house developed pharmaceutical formulations. Moreover, this study is advantageous because it protects analytical scientists and chemists from the exposure of volatile and corrosive organic solvents during experimentation and reduces the toxicity of the mobile phase making it a green analytical method by complying with the 5^th ^principle of green chemistry as proposed by Anastas and Warner (24).

## Experimental

*Chemicals and reagents*

Glibenclamide (purity 99%) was purchased Alfa Aesar, A Johnson Metthey Company (Ward Hill, MA). HPLC grade methanol and ethanol, hydrochloric acid (HCl), sodium hydroxide (NaoH) and hydrogen peroxide (H_2_O_2_) were purchased from BDH Laboratory supplies (Liverpool, UK). All other chemicals and reagents used were of analytical reagent (AR) grade. Commercial tablets of glibenclamide were purchased from local market of Riyadh, Saudi Arabia. Glibenclamide nanoemulsion was prepared in the laboratory by spontaneous emulsification method using Lauroglycol-90 as the oil phase, Labrasol, Transcutol-HP, and distilled water as surfactant, cosurfactant and aqueous phase respectively.

*Instrumentation and chromatographic conditions*

Liquid chromatographic identification of glibenclamide was performed at room temperature (25 ± 1 ^o^C), with Waters HPLC system (Waters, USA). The HPLC system was equipped with a 600 LC pump, 717 autosampler, quaternary LC-10A VP pumps, a programmable UV–visible variable-wavelength detector, SPD-10AVP column oven, a SCL 10AVP system controller (Shimadzu, Japan) and a vacuum degasser. The software used for data analysis in the system was Millennium, version 32. The identification of glibenclamide was achieved on a Lichrosphere 250 X 4.6 mm RP C_8 _column (Phenomenex, USA) having a 5 μm packing as a stationary phase. The mixture of solvents methnol: ethanol (50:50 % v/v) was used as an environmentally benign mobile phase. The elution was performed at a flow rate of 1.0 mL/min with UV detection at 245 nm. Samples (10 μL) were injected using a Waters auto sampler. 

*Preparation of drug stock solution for calibration curve*

Calibration curve for glibenclamide was prepared in the range of 0.1 to 200 μg/mL. Stock solution of 200 μg/mL concentration was prepared by dissolving 20 mg of glibenclamide in 100 mL of mobile phase. Serial dilutions from this stock solution were made by diluting the required aliquots with mobile phase to get concentration in the range of 0.1 to 200 μg/mL.

*Method development*

The criterion for selection of the solvent system was based on the sensitivity of the assay, suitability for stability studies, rapid analysis, peak parameters, miscibility, ease of preparation and finally the availability of cost effective green solvents. Based on above criterion, we had tried ethanol, ethyl acetate, ethanol–water, ethanol- methanol, glycerol, glycerol-water alone as well as at different proportions. Out of tried mobile phases for chromatographic identification, environmentally benign combination of ethanol- methanol (50:50 % v/v) was selected as final mobile phase for further studies. 

*Validation studies*

The proposed green RP-HPLC method was validated in compliance to ICH guidelines for the linearity, selectivity, accuracy, precision, sensitivity, robustness and specificity. Freshly prepared linearity solutions of different concentration (0.1-200 μg/mL) were used for construction of calibration curves. The mobile phase (ethanol- methanol 50:50 % v/v) was delivered at 1.0 mL/min for column equilibration; the baseline was monitored continuously during this process. The detection was performed at 245 nm. The prepared dilutions were injected in triplicates and peak areas were recorded for each dilution, and concentration was plotted against peak area. 

The selectivity of the developed method was determined by repeated injections of target concentration of glibenclamide (20 μg/mL). The variations in retention time and peak area were recorded for selectivity determination. 

Accuracy of the proposed method was determined in commercial tablets of glibenclamide by the standard addition method. The preanalyzed sample of glibenclamide tablets (20 μg/mL) was spiked with 0, 50, 100 and 150% extra glibenclamide tablets solution and were reanalyzed by the proposed method. Experiments were performed in triplicates at each concentration level. Recovery (%), RSD (%), and standard error for each concentration were calculated. Precision of the green HPLC method was determined as repeatability (intraday precision) and intermediate precision as per ICH guidelines. Repeatability studies were performed by analysis of four different concentrations of glibenclamide (20, 30, 40 and 50 μg/mL) in triplicate on the same day. Intermediate precision of the method was checked by repeating the studies on three different days at same concentration level. 

Detection (LOD) and quantification (LOQ) limits of the developed method were determined by the standard deviation (SD) method as reported previously (34). For determination of LOD and LOQ, blank samples (samples without glibenclamide) were injected in triplicate and the peak area of these blank samples was recorded. LOD and LOQ were determined from the slope (S) of the calibration curve and the standard deviation (SD) of the response by use of the formulae LOD = 3.3 × SD/S and LOQ = 10 × SD/S. 

The robustness of the method was determined to evaluate the effect of deliberate variation of chromatographic conditions on determination of glibenclamide. The target concentration (20 μg/mL) was selected for these studies. Robustness was determined by changing the mobile phase composition from 50:50 to 45:55 and 55: 45, flow rate from 1.0 mL/min to 0.75 and 1.25 mL/min and wavelength of detection from 245 nm to 240 and 250 nm. 

*Forced degradation studies*

In order to determine the stability-indicating property and specificity of the green HPLC method, force degradation studies were performed at various stress conditions such as acid stress, base stress, oxidative stress and thermal stress conditions. 

For acid and base-induced degradation, the target concentration (20 μg/mL) of glibenclamide was freshly prepared into mobile phase. An aliquot of this solution (20 μg/mL) was exposed to acid and base hydrolysis by adding 4 mL of 0.1 M HCl and 4 mL of 0.1 M NaOH respectively. These mixtures were kept at hot air oven for 48 h at 50 °C and then reanalyzed by developed method for determination of glibenclamide in the presence of its acid and base degradation products respectively. For oxidative degradation, an aliquot of target solution (20 μg/mL) was exposed to oxidative degradation by adding 4 mL of 30% H_2_O_2_. This mixture was kept at hot air oven for 48 h at 50 °C and then analyzed by developed method for determination of glibenclamide in the presence of its oxidative degradation products. For thermal degradation, an aliquot of target solution (20 μg/mL) was exposed to thermal degradation by exposing it at hot air oven for 48 h at 50 °C and then analyzed by above stated method for determination of glibenclamide in the presence of its thermal degradation products. 

*Solution stability studies*

To ensure the reliability of the results in relation to handling and storage of stock standards, solution stability studies were performed at target concentration (20 μg/mL) by repeated analysis of the samples over a period of 72 h at ambient temperature (25 ± 1 °C) and at the refrigerated temperature (4 ± 0.5 °C). 

*Application of proposed method for the assay of glibenclamide in nanoemulsion and commercial tablets*

Nanoemulsion formulation of glibenclamide was prepared in the laboratory by spontaneous emulsification method using Lauroglycol-90, Labrasol, Transcutol-HP and distilled water as the oil phase, surfactant, cosurfactant and aqueous phase respectively. To determine the glibenclamide content in developed nanoemulsion (containing 5 mg/mL of glibenclamide-single dose), 1 mL of nanoemulsion was suitably diluted with mobile phase to obtain 100 mL of stock solution. This solution was sonicated for 10 min and then the sample so obtained was analyzed for drug content. The possibility of interference of nanoemulsion components present in the formulation was studied. For determination of content of glibenclamide in marketed tablets, 100 mL stock of each formulation was prepared and analyzed for drug content using same procedure as used for nanoemulsion. 

## Results and Discussion

*Method development*

**Figure 2:**
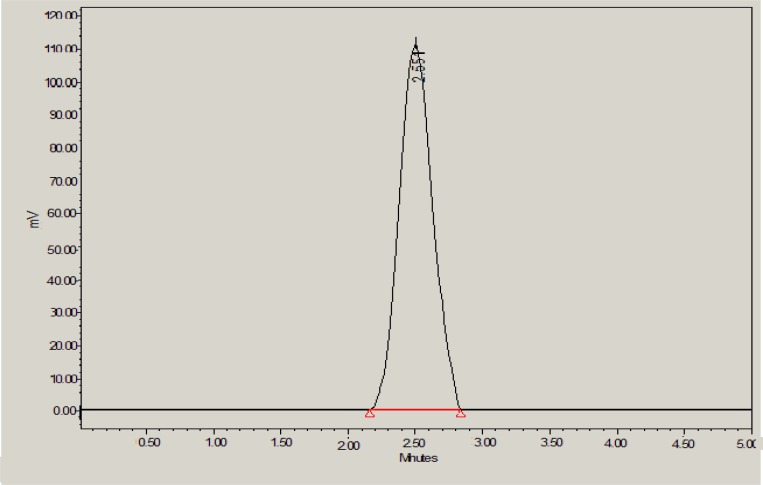
HPLC chromatogram of glibenclamide in methanol: ethanol (50:50 % v/v) with retention time of 2.551 min

With the background to develop an environmentally benign RP-HPLC method for rapid analysis of glibenclamide in developed nanoemulsion and marketed tablets, various solvent systems as mobile phase were tried. During method development step, use of ethanol or ethyl acetate or methanol or glycerol alone as the mobile phase resulted in a very poor asymmetric peak with a greater tailing factor (2.5). Further, glycerol-water, ethyl acetate-ethanol and ethanol-water were tried at different proportions at flow rate of 1.0 mL/min. Again, a chromatograph with a very poor peak with greater asymmetry was obtained. In order to get sharp peak with acceptable asymmetry factor (less than 2) and good sensitivity, ethanol-methanol as another mobile phase was tried. The wavelength of detection for quantification of glibenclamide was set at 245 nm because it is reported wavelength of glibenclamide (18-21). Combination of ethanol and methanol (50:50 % v/v) was found to be better with sharp peak, suitable retention time and good asymmetry factor (1.14). Therefore, this combination was selected as final mobile phase to obtain a rapid and simple assay method for glibenclamide with a reasonable run time (5 min), suitable retention time (2.55 ± 0.008 min) and the acceptable tailing or asymmetry factor as shown in Figure 2. 

*Method validation*

The calibration curve of pure glibenclamide was evaluated by linear least square statistical analysis. The calibration curve of peak response (peak area) versus concentration was found to be linear in the concentration range of 0.1–200 μg/mL**. **linear regression equation for the calibration curve was found to be y = 25092x+32322. The correlation coefficient (*r*^2^) for this calibration was found to be 0.999 ± 0.0007. The whole linear regression data for calibration curve of glibenclamide is shown in Table 1.

**Table 1:** Linear regression data for the calibration curves of glibenclamide (n = 3).

**Parameters**	**Values**
Linearity range	0.1– 200 μg/mL
Correlation coefficient (r^2 ^± SD)	0.999 ± 0.0007
Regression equation	Y= 25092x+32322
Slope ± SD	25092.00 ± 183.44
Confidence interval of slope*	24636.26-25547.73
Standard error of slope	105.91
Slope without intercept ± SD	25382.00 ± 195.76
Intercept ± SD	32322.00 ± 652.54
Confidence interval of intercept*	30700.82-33943.15
Standard error of intercept	376.75

The selectivity of proposed HPLC method was determined by repeated injections of glibenclamide at the concentration of 20 μg/mL. The values of SD and % RSD in peak area as well as in retention time were found to be very low (Table 2). The % RSD in peak area and retention time was found to be 0.94 and 0.19 respectively which indicated the selectivity of proposed analytical method.

**Table 2:** Selectivity of proposed HPLC method (n = 6).

**S. No.**	**Concentration (μg/mL)**	**Peak Area**	**Mean Area ± SD **	**RSD (%)**	**R**_t _ **(min)**	**Mean R**_t_ **± SD (% RSD)**
1		562876			2.55	
2		559899			2.54	
3	20	565467	559727 ± 5301	0.94	2.55	2.545 ± 0.005 (0.19)
4		557865			2.55	
5		551234			2.54	
6		561023			2.54	

The accuracy of proposed HPLC method was determined in commercial tablets as % recovery. Excellent recoveries (98.16–101.05 %) of the spiked standard were obtained at each concentration level (Table 3). The values of SD were observed as 0.31-0.75 at each concentration level. The % RSD (1.46-1.75%) and standard error (0.17-0.43) of the method were found to be very low which indicated the accuracy of the proposed method.

**Table 3:** Accuracy of proposed HPLC method (% recovery, n = 3).

**% of standard** **added to analyte**	**Theoretical concentration (μg/mL)**	**Measured concentration (μg/mL) ± SD**	**RSD (%)**	**Standard error**	**% Recovery**
0	20	20.21 ± 0.31	1.53	0.17	101.05
50	30	29.45 ± 0.43	1.46	0.24	98.16
100	40	39.38 ± 0.69	1.75	0.39	98.45
150	50	50.32 ± 0.75	1.49	0.43	100.64

The results of intraday and interday/intermediate precision were expressed in terms of % RSD (Table 4). The developed analytical method was found to be precise in terms of % RSD values for intraday and intermediate precision. % RSD values were found in the range of 1.47-1.81 and 1.29-1.61 for intraday and intermediate precision respectively. Low value of % RSD indicated the precision of the proposed analytical method. 

**Table 4:** Precision of proposed HPLC method (n = 3).

**Concentration (μg/mL)**	**Repeatability (Intra-day precision)**						**Intermediate precision (Inter-day)**		
	**Mean area ± SD**	**RSD (%)**		**Standard error**		**Mean area ± SD**		**RSD (%)**	**Standard error**
20	557865 ± 10121		1.81		5843		552342 ± 8943	1.61	5163
30	842354 ± 12456		1.47		7191		839987 ± 10899	1.29	6292
40	1010298 ± 17643		1.74		10013		998765 ± 14321	1.43	8268
50	1323124 ± 22765		1.72		13143		1298765 ± 18654	1.43	10770

LOD and LOQ for the proposed method were determined by the blank response method with the help of SD. The values of LOD and LOQ were found to be 0.12 and 0.36 μg/mL, respectively, which indicated the sensitivity of the proposed analytical method. 

For robustness of proposed analytical method, the SD, % RSD and standard error of the peak areas for mobile phase composition, wavelength of detection and flow rate at a concentration level of 20 μg/mL are shown in Table 5. The low values of % RSD and standard error were which indicated the robustness of the proposed method.

**Table 5:** Robustness of proposed HPLC method (n = 3).

**Parameters **	**Mean area ± SD **	**RSD (%) **	**Standard error **	**Retention time ± SD **	**RSD (%) **	**Standard error **
**Mobile phase composition **						
(55:45 % v/v)	548965 ± 7654	1.39	4419	2.43 ± 0.04	1.64	0.023
(45:55 % v/v)	541243 ± 6987	1.29	4034	2.62 ± 0.05	1.90	0.028
Mobile phase flow rate						
(1.25 mL/min.)	569899 ± 8098	1.42	4675	2.31 ± 0.03	1.29	0.017
(0.75 mL/min.)	532765 ± 7987	1.49	4611	2.67 ± 0.05	1.87	0.028
Detection wavelength (nm)						
240	558765 ± 7543	1.34	4355	2.56 ±0.04	1.56	0.023
250	553432 ± 7187	1.29	4149	2.57 ±0.03	1.16	0.017

*Forced degradation studies*

Glibenclamide is a hypoglycemic drug with sulphonylurea structure containing two moieties, which are susceptible to degradation at least under harsh conditions: on the one hand the benzamide group and on the other hand the sulphonylurea moiety, which can be split at the urea amide bonds. As a result, several degradation products are anticipated to be formed during formal stability testing of the drug (35).

Therefore, stability-indicating property and specificity of proposed HPLC method was determined by exposing 20 μg/mL of glibenclamide under various stress conditions. 

**Figure 3:**
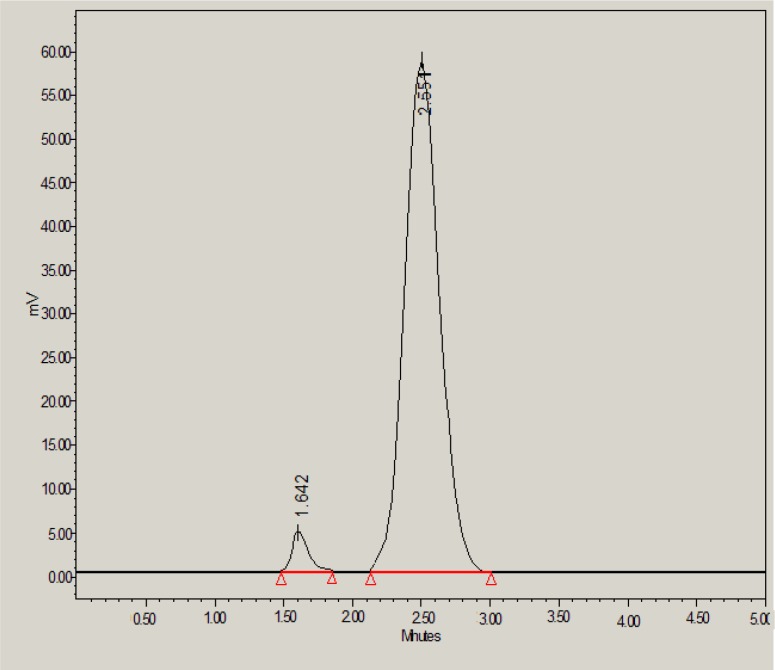
HPLC chromatogram of glibenclamide in the presence of 0.1M HCl

Results of forced degradation studies indicated that glibenclamide was found to be degraded either slowly or moderately under all stress conditions and glibenclamide peak was well resolved in the presence of all degradation products. Under acid stress conditions, 92.05% of glibenclamide was found to be remained and only 7.95% of drug was found to be degraded (Table 6 and Figure 3). The acid-induced degradation product (peak 1 in Figure 3) was found to be eluted with retention time of 1.64 min. 

**Figure 4:**
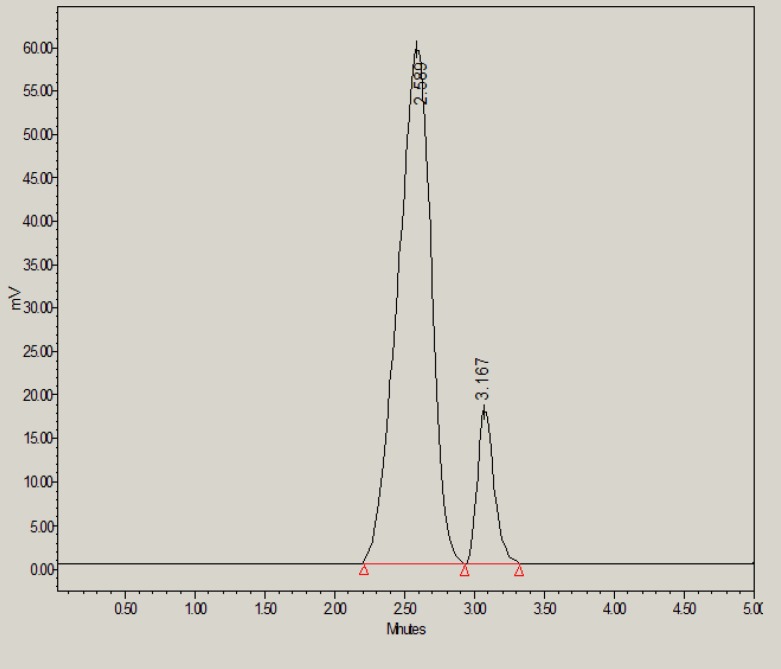
HPLC chromatogram of glibenclamide in the presence of 0.1M NaOH

However, under base stress conditions, 73.50% of glibenclamide was remaining and 26.50% was degraded (Table 6). The base-induced degradation product (peak 2 in Figure 4) was found to be eluted with retention time of 3.16 min.

**Table 6:** Forced degradation studies of glibenclamide at various stress conditions (n = 3).

**Stress condition **	**Mean area ± SD**	**RSD (%)**	**Standard error**	**Number of degradation products (R**_t_ **)**	**Glibenclamide remaining (μg/mL)**	**Glibenclamide recovered (%)**
0.1M HCl	494321 ± 5042	1.01	2911	1 (1.64)	18.41	92.05
0.1M NaOH	401278 ± 4532	1.12	2616	1 (3.16)	14.70	73.50
30 % H_2_O_2_	356496 ± 3786	1.06	2185	2 (0.74, 1.71)	12.91	64.55
Thermal	380345 ± 3986	1.04	2301	1 (1.28)	13.86	69.30

**Figure 5:**
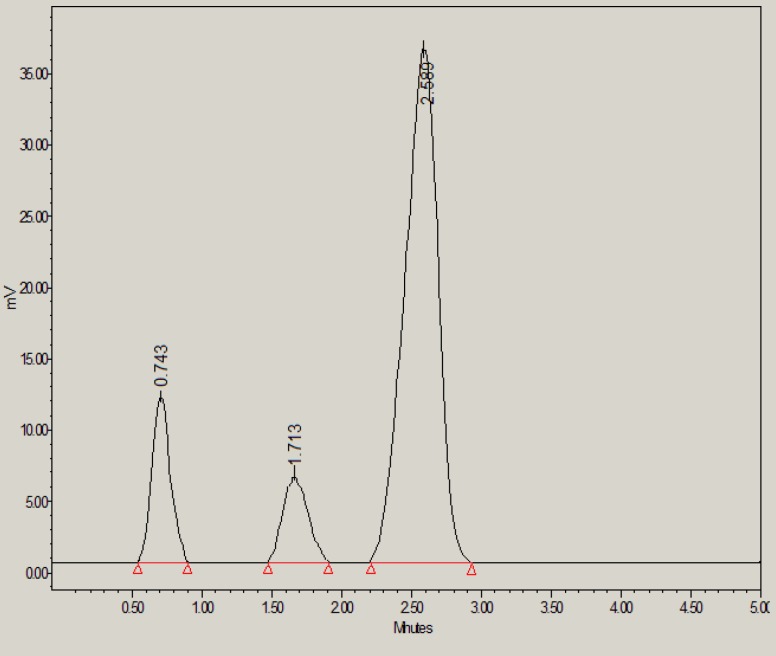
HPLC chromatogram of glibenclamide in the presence of 30% H_2_O_2_

The glibenclamide was also found to be degraded sufficiently under oxidative stress conditions (*i.e*. 30% H_2_O_2_) (Figure 5). Under oxidative stress, 64.55% of glibenclamide was remaining and 35.45% was degraded. The H_2_O_2_-induced degradation products (peak 1 and 2 in Figure 5) were found to be eluted with a retention time of 0.74 and 1.71 min respectively.

**Figure 6:**
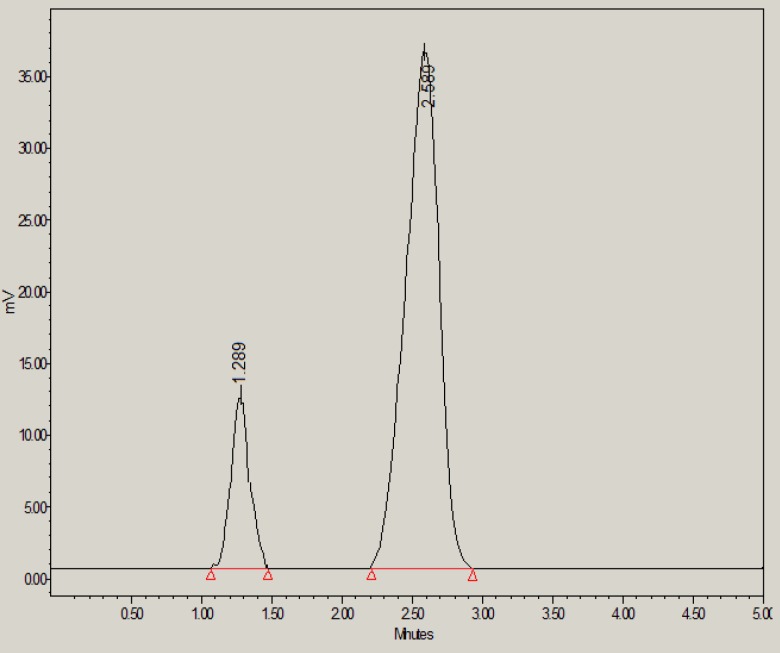
HPLC chromatogram of glibenclamide under thermal condition

However under thermal stress conditions, 69.30% of glibenclamide was remaining and 30.70% was degraded. The thermal-induced degradation product (peak 1 in Figure 6) was found to be eluted with retention time of 1.28 min. 

Overall these results indicated that proposed HPLC method was specific and stability-indicating as it resolved the glibenclamide peak in the presence of all degradation products.

*Solution stability*

Glibenclamide was found to be stable when stored for 72 h at ambient temperature (25 ± 1 °C) as well as under refrigeration (4 ± 0.5 °C) in combination of ethanol and methanol (mobile phase). More than 99% of glibenclamide was found to be unchanged, on the basis of comparison of peak areas with those obtained from a freshly prepared solution of glibenclamide. 

*Application of the proposed method for assay of glibenclamide in nanoemulsion and commercial tablets*

The proposed analytical (HPLC) method developed was found to be rapid, selective, precise, sensitive and stability-indicating for the quantification of glibenclamide. Therefore, proposed HPLC method was applied for the quantitative analysis of glibenclamide in in-house developed nanoemulsion and commercial tablets. The amount of glibenclamide in developed nanoemulsion and commercial tablets was found to be 100.50 ± 1.25 % and 99.15 ± 1.85 % respectively. High assay value of glibenclamide in nanoemulsion and commercial tablets suggested that proposed method could be suitable for routine analysis of glibenclamide in commercial pharmaceutical dosage forms. The chromatogram of glibenclamide extracted from the nanoemulsion and commercial tablets was matching with that of pure glibenclamide, showing the purity of peak in both formulations. Moreover, there was no interaction between glibenclamide and various excipients present in commercial tablets or developed nanoemulsion formulation.

## Conclusion

The proposed environmentally benign RP-HPLC method is simple, selective, rapid, accurate, precise, robust, sensitive and stability-indicating. The method was successfully applied for the assay of glibenclamide in developed nanoemulsion and commercial tablets. High assay value in case of both formulations indicate the suitability of proposed method for routine analysis of glibenclamide in commercial formulations. The method is also simple in terms of sensitivity, use of green mobile phase, rapid analysis and UV detection. The proposed method could also be applied for prediction of shelf life and half life of glibenclamide in various commercial formulations because it is having stability-indicating properties. The replacement of widely-used toxic solvents and chemicals with new, innocuous, and less toxic one provides environmentally benign alternatives to the more hazardous chemicals in the field of drug/pharmaceutical analysis. 
